# Defining safe bladder preservation in BCG-unresponsive high-risk non-muscle-invasive bladder cancer: beyond complete response

**DOI:** 10.3389/fimmu.2026.1983996

**Published:** 2026-10-22

**Authors:** Chaohu Chen, Botao Xie, Cheng Chen, Peng Gu, Xinan Chen

**Affiliations:** Second Department of Urology, The First Affiliated Hospital of Kunming Medical University, Kunming, China

**Keywords:** BCG-unresponsive, bladder preservation, complete response, non-muscle-invasive bladder cancer, radical cystectomy, treatment outcomes

## Abstract

Bacillus Calmette-Guerin (BCG)-unresponsive high-risk non-muscle-invasive bladder cancer (NMIBC) remains a challenging disease state in which radical cystectomy (RC) provides the most reliable oncologic control, while an expanding range of bladder-preserving therapies offers alternatives for patients who decline or are unfit for surgery. We critically reviewed PubMed/MEDLINE, international guidelines, pivotal and late-phase trials, long-term follow-up studies, regulatory summaries, systematic reviews, and multicenter real-world studies through 30 September 2026, prioritizing evidence relevant to patient selection, response durability, progression risk, salvage RC, treatment burden, and patient-reported outcomes. Pembrolizumab, nadofaragene firadenovec, nogapendekin alfa inbakicept plus BCG, the gemcitabine intravesical system (TAR-200), oncolytic viral approaches, FGFR-directed therapy, and sequential intravesical gemcitabine/docetaxel have broadened treatment options in this setting. However, cross-trial comparisons based on a single 3- or 6-month complete response are potentially misleading because disease phenotype, response definitions, surveillance intensity, biopsy requirements, treatment duration, and failure management differ substantially across studies; for most platforms, cross-trial efficacy estimates should therefore be interpreted descriptively rather than as head-to-head comparisons. We propose an author-derived, non-validated hierarchical framework in which clinically meaningful bladder preservation integrates phenotype-matched early tumor clearance, 12- to 24-month response durability, high-grade recurrence-free survival, freedom from muscle-invasive or metastatic progression, the timing and pathologic outcomes of subsequent RC, and treatment burden. This framework is intended to organize clinical interpretation rather than function as a validated score, guideline, or consensus recommendation. Persistent or recurrent high-grade disease, particularly T1 recurrence or suspected progression, should prompt restaging and renewed discussion of RC. Bladder preservation should thus be regarded as a time-limited, closely monitored management strategy with explicit entry criteria and stopping rules. Its success should be defined by durable high-grade disease control without compromising the opportunity for curative salvage RC, rather than by preservation of the bladder alone.

## 1 Introduction

The goal of treatment for high-risk non-muscle-invasive bladder cancer (NMIBC) is not merely to eradicate visible tumor. It is to achieve durable control while minimizing the risk of progression to muscle-invasive or metastatic disease and preserving an acceptable quality of life. Complete transurethral resection of bladder tumor (TURBT), followed by adequate bacillus Calmette-Guerin (BCG) induction and maintenance, remains the foundation of treatment for high-risk NMIBC. When high-grade disease persists or recurs after adequate BCG, major guidelines consider radical cystectomy (RC) the oncologically preferred option for patients who are fit and willing to undergo surgery (1–3). Bladder-preserving therapy is therefore most relevant to patients who decline RC or are medically unsuitable for it and who can undergo rigorous surveillance.

BCG-unresponsive disease is not simply another recurrence in which more BCG can be given without reconsidering the treatment strategy. The definition identifies patients for whom additional BCG alone has a low probability of meaningful benefit and for whom the consequences of delayed progression may be substantial. In practice, however, many patients defer or decline RC because of age, comorbidity, concerns about urinary diversion and quality of life, or personal preference. At the same time, the therapeutic landscape has expanded rapidly, creating several intravesical and systemic options with different mechanisms, schedules, toxicities, and levels of evidence (4).

BCG-failure states are clinically distinct and therapeutically heterogeneous. BCG-unresponsive disease requires adequate prior BCG exposure and identifies patients in whom further BCG alone is unlikely to provide durable benefit. In contrast, BCG-intolerant patients discontinue treatment because of toxicity before completing an adequate course and therefore may not have demonstrated biological resistance to BCG. Patients with insufficient BCG exposure likewise have not completed an adequate therapeutic course and may remain candidates for additional BCG when clinically feasible. The distinction among these groups is clearer with respect to the expected benefit of further BCG than to their observed natural-history outcomes. In a 2026 multicenter validation study, the 5-year progression rates were 29% for BCG-unresponsive disease and 30% for BCG-exposed disease after inadequate BCG. Real-world data similarly indicate a substantial burden of progression and cystectomy among BCG-exposed patients (5, 6). Dedicated outcome data for BCG-intolerant patients remain limited because intolerance is defined by inability to complete therapy rather than by a specific tumor-biological resistance phenotype. Thus, BCG-intolerant and insufficiently exposed patients should not automatically be classified as BCG-unresponsive. At the same time, incomplete BCG exposure should not be regarded as oncologically benign, and outcome data should not be extrapolated uncritically across these categories.

The clinical problem has consequently shifted. The key question is no longer whether active bladder-preserving treatments exist, but how success should be defined and how long a bladder-preserving strategy can be pursued without jeopardizing curative salvage. Recent reviews have appropriately synthesized individual modalities, including gene-mediated therapy (7), yet modality-specific efficacy summaries do not resolve the cross-platform decision problem. A high early complete response (CR) rate may be attractive, but early response is not synonymous with durable control, freedom from progression, or safe preservation of the opportunity for RC.

This review therefore evaluates contemporary bladder-preserving approaches through a urologic decision-making lens. We focus on three questions that directly affect practice: which patients can reasonably enter a bladder-preserving pathway; which outcomes justify continuation of the chosen strategy; and which events should trigger restaging and renewed consideration of RC. We propose that treatment success should be assessed hierarchically, from phenotype-matched early tumor clearance to durability, progression avoidance, salvage safety, and patient-centered value. This hierarchy is an author-proposed conceptual framework intended to organize interpretation of heterogeneous evidence; it has not been prospectively validated and should not be construed as a formal guideline, consensus statement, or scoring system.

## 2 Evidence acquisition

This narrative review was informed by searches of PubMed/MEDLINE, major urologic oncology journals, AUA/SUO, EAU, and ESMO guidelines, and peer-reviewed regulatory summaries. The literature search was updated through 30 September 2026. Search terms included “BCG-unresponsive”, “BCG-exposed”, “BCG-intolerant”, “high-risk non-muscle-invasive bladder cancer”, “bladder preservation”, “radical cystectomy”, “complete response”, “duration of response”, “pembrolizumab”, “nadofaragene firadenovec”, “nogapendekin alfa inbakicept”, “TAR-200”, “cretostimogene grenadenorepvec”, “erdafitinib”, “gemcitabine docetaxel”, “urine tumor DNA”, and “patient preference”, alone and in combination.

Potentially relevant titles and abstracts were screened for direct relevance to the review questions, and full-text articles were examined when they could meaningfully inform patient selection, staging, response assessment, progression risk, treatment burden, treatment sequencing, or salvage RC. Evidence considered eligible included international guidelines; prospective pivotal or late-phase trials and their long-term follow-up; peer-reviewed regulatory summaries; systematic reviews and meta-analyses; multicenter comparative studies; and larger real-world studies involving high-risk NMIBC after BCG or directly informing staging and salvage decisions. Preclinical studies, case reports, small single-center series when higher-level evidence was available, conference abstracts superseded by full publications, and mixed-risk or mixed-BCG-status cohorts without clinically separable outcomes were not used as primary evidence. Reference lists of key reviews, guidelines, and pivotal trials were also hand-searched to identify additional relevant studies.

Because this review was designed as a narrative rather than a systematic review, no protocol was registered, and no exhaustive database deduplication, independent dual-reviewer screening, formal risk-of-bias assessment, or PRISMA-style study-flow reporting was performed. These methodological limitations reduce reproducibility relative to a systematic review and are acknowledged explicitly. Within this narrative framework, evidence was prioritized according to clinical authority and maturity: international guidelines; pivotal or late-phase clinical trials; long-term follow-up studies; regulatory approval summaries; systematic reviews and meta-analyses; multicenter comparative studies; and real-world studies. The evidence base remains dominated by studies published from 2022 onward, with the KEYNOTE-057 cohort A report retained as a key registration study (8).

Because definitions of adequate BCG exposure, BCG-unresponsive disease, CIS cohorts, papillary-only cohorts, efficacy endpoints, biopsy requirements, denominator handling, reinduction, treatment duration, and follow-up vary substantially across studies, we deliberately avoid unadjusted ranking of treatment platforms by CR or recurrence-free rates. **Table 1** is therefore intended as a descriptive overview rather than a comparative efficacy ranking. Similarly, the hierarchical endpoint framework in **Table 2** is an author-proposed conceptual synthesis intended to structure interpretation and shared decision-making. It has not been formally validated and should not be interpreted as a guideline or consensus recommendation. Evidence-based statements in this review are grounded in cited guidelines and clinical studies, whereas the proposed endpoint hierarchy and the integration of entry criteria and stopping rules are explicitly presented as the authors’ synthesis.

**Table 1:** Contemporary bladder-preserving strategies for BCG-unresponsive high-risk NMIBC.

Treatment platform	Representative evidence	Best-matched phenotype	What to evaluate	Key limitations
Pembrolizumab	KEYNOTE-057 (8, 12)	CIS +/- papillary tumors; high-grade Ta/T1 without CIS	Phenotype-matched CR or DFS; DOR; systemic immune toxicity	Systemic adverse events; not all early responses are durable
Atezolizumab	SWOG S1605 (13)	BCG-unresponsive high-risk NMIBC	6-month CR; durability; systemic toxicity	Modest activity; class effects should not be assumed
Nadofaragene firadenovec	Phase 3 study and 5-year follow-up (14–16)	CIS +/- papillary tumors	Durable CR; HG-RFS; timing of RC	Intravesical procedures and continued intensive surveillance
NAI + BCG	QUILT-3.032 (17, 18)	CIS +/- papillary tumors	Durable response; dependence on BCG re-exposure; completion	BCG availability, tolerance, and repeated catheterization
Gemcitabine intravesical system (TAR-200/Inlexzo)	SunRISe-1 and regulatory approval (19, 20)	CIS +/- papillary tumors	Durability; device tolerance; reproducibility; salvage timing	Device exchange, urinary symptoms, infection, resource use
Cretostimogene monotherapy/+ pembrolizumab	CORE-001 (22); BOND-003 (23)	CIS +/- papillary tumors	CR/DOR; HG-RFS; progression; incremental combination benefit	Single-arm phase 3/2 evidence; comparative and long-term salvage data maturing
Erdafitinib	THOR-2 (24)	FGFR-altered papillary-only disease	RFS; molecular selection; systemic safety	Applicable only to molecularly selected patients
Sequential gemcitabine/docetaxel	Comparative and multicenter real-world studies (25–27)	Post-BCG high-risk NMIBC in patients avoiding RC	HG-RFS; standardized regimen; subsequent RC	Less mature prospective registration evidence

BCG, bacillus Calmette-Guerin; CIS, carcinoma in situ; CR, complete response; DFS, disease-free survival; DOR, duration of response; FGFR, fibroblast growth factor receptor; HG-RFS, high-grade recurrence-free survival; NAI, nogapendekin alfa inbakicept; NMIBC, non-muscle-invasive bladder cancer; RC, radical cystectomy; RFS, recurrence-free survival. Regulatory approval and access vary by jurisdiction.

**Table 1** is intended as a descriptive overview of contemporary treatment options rather than an efficacy ranking. Differences in disease phenotype, prior BCG exposure, response-assessment timing, mandatory-biopsy requirements, handling of unevaluable patients, treatment duration, follow-up, and regulatory status limit the validity of unadjusted cross-trial comparisons.

**Table 2:** Author-proposed, non-validated hierarchical endpoint framework for evaluating bladder-preserving treatment.

Level	Clinical question	Preferred endpoints	Clinical interpretation
1. Early effect	Has the treatment produced an initial antitumor effect?	CIS: 3- or 6-month CR; papillary disease: early HG-RFS/DFS	Supports continuation; does not by itself define success
2. Durable control	Can the initial benefit be maintained?	12- and 24-month sustained CR; DOR; HG-RFS	Closer to meaningful long-term benefit than a single time-point CR
3. Progression avoidance	Does treatment prevent crossing the curable boundary?	Freedom from MIBC; metastasis-free survival; DSS/OS	Separate low-grade recurrence, high-grade recurrence, and true progression
4. Salvage safety	Can failure still be treated with timely RC?	Subsequent RC rate and timing; pathologic stage; nodes; margins; perioperative outcomes	Prevents unsafe delay of RC from being mislabeled as bladder-preservation success
5. Patient-centered value	Is the treatment acceptable and realistically deliverable?	PROs; quality of life; urinary symptoms; treatment completion; visits; economic burden	Determines real-world feasibility and quality of shared decision-making

CIS, carcinoma in situ; CR, complete response; DFS, disease-free survival; DOR, duration of response; DSS, disease-specific survival; HG-RFS, high-grade recurrence-free survival; MIBC, muscle-invasive bladder cancer; OS, overall survival; PRO, patient-reported outcome; RC, radical cystectomy.

This framework represents an author-derived synthesis intended to support interpretation and shared decision-making. It is not a formally validated scoring system, guideline, or consensus recommendation. Its individual components should therefore be interpreted in the context of disease phenotype and the strength of the supporting evidence.

## 3 Before bladder preservation: confirm phenotype and the cystectomy boundary

### 3.1 Confirm the BCG-failure state and complete staging

Before a patient is labeled BCG-unresponsive, adequate prior BCG exposure should be confirmed. In general, adequate BCG comprises at least five of six induction instillations plus at least two of three maintenance instillations or at least two of six doses of a second induction course. High-grade Ta/T1 recurrence within 6 months after adequate BCG, persistent or recurrent carcinoma *in situ* (CIS) within 12 months, or high-grade T1 disease at the first evaluation after adequate BCG identifies a population in whom further BCG alone should not be the default strategy (1, 2). BCG intolerance, inadequate BCG exposure, and late relapse outside these windows are clinically distinct states, and evidence should not be extrapolated across these categories without caution.

The first step in bladder preservation is not drug selection; it is confirmation that the disease remains within a safely salvageable NMIBC state. Visible papillary tumors should be completely resected whenever feasible. Repeat TURBT, enhanced cystoscopy, directed or mapping biopsies, prostatic urethral sampling, and upper urinary tract assessment should be considered when there is high-grade T1 disease, absent muscularis propria in the initial specimen, suspected residual disease, variant histology, lymphovascular invasion, possible prostatic urethral involvement, unexplained positive cytology, or suspicious imaging (1, 2). Evidence of muscle-invasive disease, regional nodal involvement, or distant metastasis should end a bladder-preserving pathway predicated on NMIBC.

When clinically indicated staging cannot be completed because repeat TURBT with adequate muscularis propria sampling, targeted or mapping biopsy, prostatic urethral evaluation, upper-tract assessment, or appropriate cross-sectional imaging is unavailable, the resulting uncertainty becomes an important safety concern. A contemporary meta-analysis showed that, even in the modern era, residual tumor is detected in approximately one-third of patients with T1 disease at repeat TURBT, and clinically relevant occult upstaging persists (9). Incomplete staging may therefore lead to occult MIBC or urothelial disease outside the bladder being misclassified as bladder-confined NMIBC, inappropriately expand eligibility for conservative treatment, and delay definitive surgery. When a clinically indicated staging procedure cannot be completed locally, referral to a center capable of completing the work-up should be considered. If adequate staging remains unattainable, the threshold for recommending RC rather than prolonged empiric bladder-preserving treatment should be lower, particularly in patients with high-grade T1 disease, variant histology, lymphovascular invasion, unexplained positive cytology, or suspected prostatic urethral or upper-tract involvement (1, 2, 9, 10).

### 3.2 Match disease phenotype to response assessment and the cystectomy boundary

Disease phenotype must also determine the efficacy language. CIS is flat, often multifocal, and may be occult at white-light cystoscopy; response assessment therefore requires cystoscopy and urine cytology, with biopsy when clinically indicated. By contrast, papillary high-grade Ta/T1 tumors are usually resected before treatment and are more appropriately evaluated by high-grade recurrence-free survival (HG-RFS), disease-free survival, or related time-to-event endpoints. Combining CIS and completely resected papillary disease into a single CR metric conflates two different questions: whether existing disease has been eradicated and whether new high-grade disease has recurred.

RC should be discussed at the time bladder preservation is first considered rather than introduced only after multiple treatment failures. The threshold for early RC should be particularly low in recurrent high-grade T1 disease, variant histology, lymphovascular invasion, prostatic urethral involvement, extensive or incompletely resectable tumors, or circumstances in which intensive surveillance cannot be reliably maintained (1, 2, 10, 11). Bladder preservation is clinically defensible only while the opportunity for potentially curative surgery is preserved.

## 4 Contemporary bladder-preserving options and their clinical positioning

### 4.1 Systemic immune-checkpoint inhibition

KEYNOTE-057 established pembrolizumab as a systemic option for patients with BCG-unresponsive high-risk NMIBC with CIS, with or without papillary tumors, who decline or are ineligible for RC (8). The study demonstrated that systemic immune-checkpoint inhibition can produce clinically meaningful CRs, while also showing that a substantial proportion of early responders later recur. Cohort B, which enrolled patients with high-grade Ta/T1 disease without CIS, appropriately used disease-free survival rather than CR as the central efficacy language (12). Together, the two cohorts illustrate why endpoint selection must follow disease phenotype rather than convenience.

The SWOG S1605 experience with atezolizumab showed more modest overall activity and clinically relevant systemic immune-related adverse events (13). These findings argue against treating immune-checkpoint inhibitors as interchangeable members of a single therapeutic class in localized bladder disease. For a disease still confined to the urothelium, the benefit of systemic therapy must be weighed against endocrine, pulmonary, hepatic, dermatologic, and other immune-mediated toxicities. Pembrolizumab is therefore most rational when systemic treatment is acceptable to the patient and when a suitable local therapy is unavailable, contraindicated, or less aligned with patient preference.

The apparent numerical difference between pembrolizumab and atezolizumab should not be interpreted as evidence that PD-1 blockade is intrinsically more effective than PD-L1 blockade in localized NMIBC. KEYNOTE-057 assessed CR at the first disease evaluation at 3 months, whereas SWOG S1605 used pathologic CR at 6 months, with mandatory biopsy, as its primary CIS endpoint and handled unevaluable patients conservatively. In an unplanned 3-month analysis, the clinical CR rate with atezolizumab was 43%, similar to the 41% 3-month CR reported with pembrolizumab. The SWOG S1605 report also noted broadly similar 12-month response estimates across the two studies (8, 13). Differences in assessment timing, biopsy requirements, denominator handling, cohort composition, and follow-up may therefore account for a substantial part of the apparent efficacy discrepancy. Biological differences between PD-1 and PD-L1 blockade cannot be excluded, but they cannot be established from these nonrandomized cross-trial comparisons.

### 4.2 Intravesical gene therapy: nadofaragene firadenovec

Nadofaragene firadenovec uses a nonreplicating adenoviral vector to deliver interferon alpha-2b to the urothelium. Five-year follow-up of the phase 3 study extended the evidence base beyond an early CR by reporting long-term high-grade recurrence, bladder preservation, subsequent RC, and survival outcomes (14). Exploratory analyses linking anti-adenovirus antibodies to response durability suggest that host immune biology may influence benefit, although these data are not sufficient for routine patient selection (15). Regulatory review has further clarified the eligible population, administration pathway, and safety profile (16).

The practical strengths of this approach are local administration, a relatively infrequent dosing schedule, and mature prospective evidence. Its limitations include the need for repeated intravesical procedures, local urinary symptoms, continued cystoscopic and pathologic surveillance, and the possibility of recurrence despite an initial CR. As with all bladder-preserving treatments, clinicians should define in advance how persistent CIS, early high-grade recurrence, and suspected progression will be managed.

### 4.3 IL-15 superagonist plus BCG

Nogapendekin alfa inbakicept (NAI; N-803) is an IL-15 superagonist that enhances natural killer-cell and CD8+ T-cell activity and is administered intravesically with BCG. QUILT-3.032 demonstrated substantial and durable response activity in BCG-unresponsive CIS (17), and subsequent regulatory review supported its use in this setting (18).

The clinical value of NAI cannot be separated from BCG re-exposure. Patients with severe prior BCG intolerance, active infection, limited bladder capacity, or inability to undergo repeated catheterization may be poor candidates. BCG availability, ability to complete maintenance therapy, and institutional logistics also matter. A high response rate should not be interpreted independently of the practical burden required to deliver the regimen.

### 4.4 Sustained intravesical gemcitabine delivery

The gemcitabine intravesical system, developed as TAR-200 and approved in the United States as Inlexzo, provides sustained local gemcitabine exposure rather than a brief dwell-time instillation. SunRISe-1 demonstrated high and durable antitumor activity in BCG-unresponsive high-risk NMIBC with CIS (19). The US Food and Drug Administration approved the system on 9 September 2025 for adults with BCG-unresponsive NMIBC with CIS, with or without papillary tumors (20). The platform is important not only because of gemcitabine itself, but because continuous local delivery changes intravesical pharmacokinetics and may improve the depth and duration of exposure.

Its clinical value must nevertheless include device-related burden. Placement and exchange procedures, urinary symptoms, infection, device expulsion or removal, outpatient resource use, and patient acceptance are part of the treatment effect in routine practice (19, 20). The registration experience also illustrates a broader challenge in BCG-unresponsive NMIBC: single-arm pivotal trials are feasible and often necessary, but as the number of active alternatives increases, external comparability, post-failure management, and long-term progression risk require progressively more scrutiny (21). Regulatory status and access vary by jurisdiction.

Device-related tolerability is also important for determining whether sustained intravesical therapy can be maintained over time rather than merely initiated successfully. In the TAR-200 monotherapy CIS cohort of SunRISe-1, treatment-related adverse events led to treatment interruption in 31.8% of patients and permanent discontinuation in 3.5%; most interruptions involved only one or two missed doses, and most affected patients subsequently resumed therapy (19). Nevertheless, recurrent lower urinary tract symptoms, infections, difficult device exchanges, early device removal, and repeated interruptions may increase the burden of follow-up, create gaps in scheduled drug exposure, and reduce patients’ willingness to continue maintenance therapy. Long-term studies should therefore report completion of the intended device dwell time, missed or delayed exchanges, unscheduled removals, treatment interruptions, permanent discontinuation, and whether device intolerance may interfere with surveillance or contribute to earlier transition to another therapy or RC (19, 20).

### 4.5 Oncolytic viral and molecularly selected approaches

Cretostimogene grenadenorepvec is an oncolytic adenovirus engineered for tumor-selective replication and GM-CSF expression. CORE-001 combined intravesical cretostimogene with pembrolizumab and reported encouraging activity in BCG-unresponsive CIS (22). The biological rationale for local oncolysis plus systemic immune activation is compelling, but the sample size, single-arm design, and dual-treatment burden limit cross-platform comparisons. Future development should distinguish the incremental contribution of local monotherapy, systemic combination therapy, and treatment sequencing.

Evidence for cretostimogene has advanced beyond the earlier CORE-001 combination study. In the single-arm phase 3 BOND-003 Cohort C, intravesical cretostimogene monotherapy achieved a centrally confirmed CR at any time in 83 of 110 evaluable patients (75%) after a median follow-up of 25.8 months. Treatment-related adverse events were predominantly low grade, with no grade 3–4 treatment-related events or treatment-related discontinuations reported (23). These findings strengthen the prospective evidence base for cretostimogene monotherapy. However, the absence of a randomized comparator and the still-maturing long-term data on progression, salvage RC, and survival limit the maturity of the evidence relative to platforms with longer regulatory experience and follow-up.

THOR-2 demonstrated that molecular selection can also enter the NMIBC pathway. In patients with FGFR-altered, papillary-only high-risk NMIBC recurring after BCG, erdafitinib improved recurrence-related outcomes (24). This represents a shift from histology-only stratification toward biologically selected treatment. However, ocular, phosphate-metabolism, and other systemic toxicities require careful monitoring, and FGFR alteration should be viewed as treatment-selection information rather than a reason to reduce staging or surveillance intensity.

### 4.6 Sequential intravesical gemcitabine/docetaxel

Sequential intravesical gemcitabine/docetaxel (Gem/Doce) has become a widely used post-BCG bladder-preserving regimen because both drugs are accessible, costs are comparatively manageable, and the delivery pathway is familiar to urologic practices. Comparative data in BCG-unresponsive NMIBC support clinically meaningful oncologic activity (25), while broader high-risk NMIBC cohorts have also shown favorable outcomes relative to BCG in retrospective comparisons (26). European multicenter experience supports cross-institutional feasibility, although nonrandomized design and follow-up duration limit certainty (27).

The major strength of Gem/Doce is pragmatic reproducibility. Its main limitation is the absence of prospective registration evidence comparable with newer approved platforms. Protocols should therefore standardize induction, maintenance, response assessment, and failure definitions. Ease of access should not become a reason to prolong ineffective therapy in persistent high-grade disease or recurrent T1 disease. The contemporary bladder-preserving strategies discussed above are summarized in **Table 1**, with emphasis on phenotype matching, response durability, treatment burden, and the implications for timely salvage RC.

Evidence maturity remains uneven across bladder-preserving platforms. Pembrolizumab, nadofaragene firadenovec, and NAI plus BCG are supported by prospective registration data and/or longer-term follow-up (8, 14, 17, 18). TAR-200 has strong prospective phase IIb evidence and U.S. regulatory approval, but, as a newer platform, long-term data on progression and salvage outcomes remain less mature (19, 20). Cretostimogene is now supported by phase 3 single-arm evidence (23), whereas Gem/Doce continues to rely predominantly on retrospective comparative studies and multicenter real-world data (25–27). Evidence for biomarker-guided treatment selection is less mature. Molecular alterations such as FGFR can identify eligibility for targeted therapy, but no urinary or circulating biomarker has yet been prospectively validated to select among bladder-preserving platforms or to justify reducing standard surveillance intensity (24, 28–31). These distinctions reflect differences in evidence maturity rather than comparative efficacy, because the available platforms have not been evaluated within a common randomized head-to-head framework.

## 5 Defining treatment success: beyond a single complete response

### 5.1 Early tumor clearance is a continuation signal, not final success

For CIS-containing disease, CR at 3 and 6 months remains an important measure of initial antitumor activity. For completely resected papillary high-grade Ta/T1 disease, early HG-RFS or disease-free survival is more informative. A negative early assessment supports continuation of an effective and tolerable therapy, but it does not establish long-term oncologic benefit. A regimen that produces many 3-month CRs but loses most responses during the subsequent year may defer a decision without meaningfully altering the disease trajectory.

Trials and clinical programs should therefore report the CR denominator, method of confirmation, biopsy policy, duration of response (DOR), treatment discontinuation, subsequent therapies, and management of recurrence. Excluding patients who do not complete evaluation, switch therapy, or proceed to RC can substantially overestimate real-world value.

### 5.2 Durability and avoidance of progression should outrank a single time-point response

Twelve- and 24-month sustained CR, DOR, HG-RFS, freedom from muscle-invasive progression, and metastasis-free survival are closer to what patients and clinicians ultimately seek than a single early CR. Outcomes should distinguish low-grade recurrence, high-grade Ta recurrence, T1 recurrence, muscle-invasive progression, upper-tract recurrence, and prostatic urethral involvement because these events have very different implications for salvage.

Cystectomy-free survival should not be interpreted alone. A retained bladder may reflect successful disease control, but it may also reflect comorbidity that precludes surgery or an unsafe delay in RC. Comparative long-term data suggest that carefully selected patients can achieve meaningful intermediate-term control with bladder-preserving treatment, while recurrence, progression, and later cystectomy continue to accumulate over time (32). Heterogeneous real-world treatment sequences reinforce the need to interpret bladder preservation together with treatment exposure, time to failure, and the subsequent salvage pathway (33).

### 5.3 Salvage radical cystectomy is a core endpoint of bladder preservation

A successful bladder-preserving strategy must maintain the option of timely salvage RC. Studies should report the proportion undergoing subsequent RC, time from the first BCG-unresponsive event to surgery, pathologic stage at RC, nodal status, margin status, perioperative outcomes, and disease-specific survival. Data in very-high-risk NMIBC indicate that selected patients may undergo a conservative strategy with acceptable outcomes under close surveillance, but these findings should not be generalized to all high-risk phenotypes (11). Variant histology is associated with upstaging and adverse outcomes and should lower the threshold for RC (10).

Baseline features associated with a higher risk of pathologic upstaging at salvage RC include high-grade T1 disease, concomitant CIS, variant histology, lymphovascular invasion, prostatic urethral or urethral involvement, multifocal or bulky disease, incomplete resection, absence of muscularis propria, and residual T1 disease at restaging TURBT (1, 2, 9, 10, 34). In a contemporary RC cohort, concomitant CIS, variant histology, and urethral involvement were independently associated with upstaging from clinically NMIBC to pathologic MIBC (34). Retrospective studies in BCG-unresponsive populations also suggest that adverse pathologic findings are more frequently observed among patients who undergo delayed cystectomy after multiple bladder-preserving treatment lines, supporting caution against serial salvage as a default strategy (32). These features do not mandate immediate RC in every patient, but they may justify a shorter interval for reassessment and more explicit counseling that salvage RC may reveal more advanced disease than suggested by clinical staging.

The clinically meaningful endpoint is therefore not simply “bladder intact”. Bladder preservation is successful only when disease remains within a potentially curable boundary and the patient retains an acceptable opportunity for salvage surgery if the chosen therapy fails.

### 5.4 Patient-centered value determines whether a strategy is truly deliverable

Patients with high-risk NMIBC must weigh more than tumor response. Repeated catheterization and cystoscopy, urinary symptoms, systemic toxicity, visit frequency, uncertainty about recurrence, effects on work and caregivers, cost, and the possibility of eventual RC all influence treatment value. Qualitative studies identify clarity of information and predictability of the disease trajectory as important unmet needs (35). Cross-country preference studies show that patients are willing to trade efficacy, toxicity, route of administration, and delay of RC, but generally place high value on avoiding progression and preserving the bladder (36). Patient preference studies also suggest that diagnostic certainty may outweigh reductions in procedural burden when considering surveillance alternatives (37).

Patient-reported outcomes (PROs), quality of life, urinary symptoms, treatment completion, unplanned visits, hospitalizations, and financial burden should therefore be treated as core rather than accessory outcomes. A therapy with biological activity that cannot be completed by a substantial proportion of patients may have less real-world value than its response rate suggests. Taken together, bladder-preserving treatment success should be evaluated hierarchically, extending from early antitumor activity to durable disease control, progression avoidance, salvage safety, and patient-centered value, as summarized in **Table 2**.

## 6 Clinical implementation: from risk confirmation to predefined stopping rules

A practical pathway should be risk layered rather than organized as a list of drugs. First, confirm that the patient truly meets criteria for BCG-unresponsive disease and exclude understaging. Second, define the current phenotype: CIS, papillary high-grade Ta, or T1 recurrence, and document variant histology, lymphovascular invasion, prostatic urethral involvement, and upper-tract risk. Third, assess surgical fitness and patient values while making clear that declining RC does not justify indefinite continuation of ineffective therapy. Fourth, select a treatment platform according to prior BCG tolerance, immune contraindications, FGFR status, bladder capacity, catheterization feasibility, infection history, local expertise, treatment access, and patient burden.

Stopping rules should be defined before treatment begins. High-grade T1 recurrence during therapy, suspected or confirmed muscle-invasive disease, nodal or metastatic progression, high-grade upper-tract or prostatic urethral disease, repeated early high-grade recurrences, or evidence that the patient is approaching loss of a safe surgical window should trigger restaging and a strong recommendation to revisit RC. Persistent CIS or high-grade Ta at the first formal assessment should also trigger explicit reassessment; protocol-specific reinduction may be reasonable in selected patients when supported by the regimen and when T1 or more advanced disease has been excluded, but persistence should not be allowed to drift into serial unstructured salvage attempts.

Surveillance intensity should not be reduced simply because a novel therapy has been chosen. Cystoscopy, urine cytology, biopsy when indicated, and risk-adapted upper-tract imaging remain the foundation. Urinary biomarkers show substantial heterogeneity in diagnostic performance and currently serve best as adjuncts rather than replacements for cystoscopic or histologic confirmation in the BCG-unresponsive setting (29, 38). Urine tumor DNA can stratify recurrence risk after immune-checkpoint therapy (30), and circulating tumor DNA in blood and urine has promise for minimal residual disease assessment (31). However, a negative molecular test is not sufficient evidence to prolong ineffective therapy or defer RC in the presence of concerning clinical or pathologic findings.

## 7 Future research priorities

The next generation of studies should move beyond another single-arm early CR rate and generate evidence that is comparable across the entire treatment pathway. CIS and papillary-only cohorts should be designed and reported separately, and BCG-unresponsive, BCG-exposed, BCG-intolerant, and late-relapsing disease should not be merged. As multiple therapeutic platforms coexist, standardized disease-state definitions, phenotype-specific endpoints, and comparative trial designs should be prioritized where feasible (39). Salvage RC should be treated as a core endpoint rather than an event occurring outside the study after protocol discontinuation.

Treatment sequencing is another major unanswered question. It remains uncertain whether a second bladder-preserving therapy after failure of the first can be delivered without increasing the risk of pathologic upstaging, which local and systemic therapies should be combined, and which recurrence patterns indicate that further conservative therapy has become oncologically unreasonable. These questions will become more important as patients gain access to multiple active platforms.

The BCG-unresponsive population itself is likely to change. In BCG-naive high-risk NMIBC, the CREST trial showed improved event-related outcomes with sasanlimab plus BCG (40), while POTOMAC supported the addition of durvalumab to BCG (41). These first-line changes may reduce, delay, or biologically reshape the patients who later meet BCG-unresponsive criteria. Contemporary reviews of the evolving BCG-naive landscape similarly indicate that immune combinations, maintenance strategies, delivery platforms, and patient selection will increasingly influence subsequent treatment pathways (42, 43).

Finally, future trials should integrate PROs, treatment burden, and health-economic outcomes with oncologic endpoints from the outset. For patients, preserving the bladder and avoiding progression remain central goals, but the process used to achieve those goals must be tolerable, monitorable, and compatible with timely salvage if the disease escapes control.

## 8 Conclusions

Bladder-preserving therapy for BCG-unresponsive high-risk NMIBC has moved from a small set of empiric salvage options to a multiplatform treatment landscape. This expansion is clinically important, but it does not change the fundamental oncologic boundary: RC remains the preferred curative strategy for eligible patients at highest risk, and any conservative pathway must begin with accurate restaging, phenotype definition, and an explicit discussion of the surgical alternative.

Treatment success should not be defined by a single 3-month CR. A clinically meaningful definition combines confirmed early tumor clearance, sustained high-grade control over at least 12–24 months, avoidance of muscle-invasive and metastatic progression, preservation of timely salvage RC, and a treatment process that patients can tolerate and complete. Applying these dimensions within an explicitly author-proposed, nonvalidated framework may help reduce misleading cross-trial comparisons, avoid misclassifying an unsafe delay in RC as successful bladder preservation, and provide a more clinically useful basis for evaluating rapidly expanding treatment options.
